# Neighborhood Environment, Intrinsic Capacity and 4-Year Late-Life Functional Ability Trajectories

**DOI:** 10.1093/geroni/igab046.1808

**Published:** 2021-12-17

**Authors:** Shiyu Lu, Yuqi Liu, Yingqi Guo, Hung Chak Ho, Hiu Kwan Chui, Chris Webster, Lai Har Chiu, Terry Y S Lum

**Affiliations:** The University of Hong Kong, Hong Kong, Hong Kong

## Abstract

Knowledge on how intrinsic capacity (IC) and neighbourhood physical environment shape functional ability (FA) trajectories in later life remains understudied. We investigated the 4-year trajectories of IC and their impact on FA trajectories, and the associations between neighbourhood physical environments and FA trajectories over time among older adults. We conducted a four-wave longitudinal study from 2014-2017 in Hong Kong with 2,081 adults aged 65 and above. FA was assessed by The Chinese Lawton Instrumental Activities of Daily Living. We used cognition, affect, locomotion, sensory capacity, and vitality to capture multi-domains of IC. Neighbourhood physical environment attributes included green space, land use diversity, and facilities availability, assessed within 200- and 500-meter buffers of respondents' homes. IC and FA each decreased significantly over time. Individuals with declines in IC experienced faster declines in FA over time. Green space, the number of leisure facilities and public transport slowed the decreasing FA rate.

